# Appropriate use of the fixed-dose, extended-release combination of naltrexone and bupropion as treatment for obesity in primary care

**DOI:** 10.1016/j.obpill.2025.100170

**Published:** 2025-02-26

**Authors:** Ethan Lazarus

**Affiliations:** Clinical Nutrition Center, Greenwood Village, CO, 80111, USA

**Keywords:** Fixed-dose, Extended-release naltrexone-bupropion, Obesity, Obesity management medication

## Abstract

**Background:**

Obesity is considered a chronic disease and is influenced by biological, environmental, and behavioral factors that can contribute to its progression. Although lifestyle changes are integral to treating obesity and maintaining a healthful weight, weight reduction from behavioral intervention alone is often insufficient because neurophysiologic factors may work against such changes in lifestyle and behavior. Research suggests that the mechanisms underlying food cravings and obesity overlap with dopaminergic signaling in the brain and pathways involved in addiction. As a result, patients who are differentially impacted by food cravings may have better outcomes with treatments targeting neural systems implicated in both homeostatic and hedonic food consumption or addictive behaviors.

**Methods:**

In this clinical review, we describe the safety and efficacy data for the fixed-dose, extended-release combination of naltrexone and bupropion (NB-ER) compared with its monotherapy constituents (naltrexone and bupropion), as well as discuss the appropriate use of NB-ER to treat patients with obesity.

**Results:**

NB-ER is approved for the treatment of patients with obesity, with studies showing that patients can achieve significant weight reduction compared with placebo when treatment is combined with a reduced-calorie diet and increased physical activity. Across NB-ER phase 3 trials, responders to treatment had a mean body weight reduction of 11.7 % at 56 weeks. Of note, the unique combination of naltrexone, an opioid receptor antagonist, and bupropion, a norepinephrine-dopamine reuptake inhibitor associated with stimulating pro-opiomelanocortin cells (POMC), in NB-ER may work together to target POMC cells to prevent endogenous negative feedback, thereby decreasing appetite and improving weight-related outcomes.

**Conclusions:**

Unlike monotherapy with its component drugs, NB-ER is optimized for the treatment of obesity. The appropriate use of NB-ER should consider the specific characteristics and adiposity-related complications of an individual.

## Graphical abstract

**Figure:**
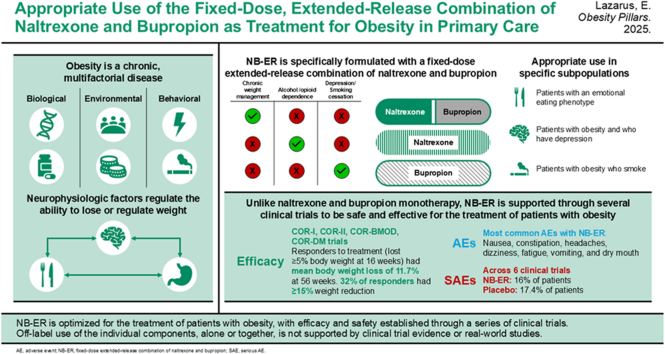

## Teaser

In the US, approximately 42.5 % of adults have obesity (body mass index [BMI], ≥30 kg/m^2^) and 31.1 % have overweight (BMI, ≥25–29.9 kg/m^2^). As a chronic disease, obesity is influenced by biological, environmental, and behavioral factors contributing to its progression. Due to neurophysiologic factors working against behavioral interventions, weight reduction from lifestyle changes alone is often insufficient. With research suggesting that the mechanisms underlying food cravings and obesity overlap with dopaminergic signaling and addiction-related brain pathways, patients impacted by food cravings may benefit from treatments that target the neural systems implicated in both homeostatic and hedonic food consumption. The unique combination of naltrexone and bupropion in the fixed-dose, extended-release combination of naltrexone and bupropion (NB-ER) may work by targeting areas of the brain involved in the control of food intake to improve weight-related outcomes. The use of NB-ER, approved for the treatment of patients with obesity, is supported by studies reporting that patients can achieve significant weight reduction (vs placebo) when treatment is combined with a reduced-calorie diet and increased physical activity. This clinical review outlines the safety and efficacy of NB-ER vs its monotherapy constituents (naltrexone and bupropion) and discusses appropriate use of NB-ER in the treatment of patients with obesity.

## 1 Introduction

According to the National Center for Health Statistics, approximately 42.5 % of adults in the United States have obesity (body mass index [BMI], ≥30 kg/m^2^) and another 31.1 % of adults have overweight (BMI, ≥25–29.9 kg/m^2^) [1]. Obesity is considered a chronic disease and is influenced by biological (genetics, gut-brain axis, medical problems, obesogenic medications, prenatal/pregnancy environment, physical disability), environmental (socioeconomic status, culture), and behavioral (chronic stress, lifestyle, level of physical activity, sleep deprivation, smoking cessation) factors that can contribute to its progression [2,3].

Obesity commonly presents with adiposity-related complications and risk factors, including major depressive disorder, cancer, cardiovascular disease, and diabetes [4,5]. Although lifestyle changes are integral to treating obesity and maintaining a healthful weight, weight reduction from behavioral intervention alone is often insufficient because neurophysiologic factors may work against such changes in lifestyle and behavior [6]. For example, patients attempting to achieve a reduced caloric intake and increased energy expenditure are often challenged by cravings [7,8]. Cravings occur spontaneously and are associated with a decreased ability to maintain long-term weight reduction [7]. Research suggests that the mechanisms underlying food cravings and obesity overlap with dopaminergic signaling centers in the brain and pathways involved in addiction [6,9]. Some treatments for obesity target neural systems that are implicated in both homeostatic and hedonic food consumption and addictive behaviors [10].

The fixed-dose, extended-release combination of naltrexone and bupropion (NB-ER) was approved by the US Food and Drug Administration (FDA) in 2014 for chronic weight management in adult patients with overweight (BMI, ≥27 kg/m^2^) and a weight-related comorbidity and patients with obesity [11]. In previous studies, patients taking NB-ER achieved significant weight reduction compared with placebo when treatment was combined with a reduced-calorie diet and increased physical activity [[12], [13], [14]]. The unique combination of naltrexone and bupropion in NB-ER may work by targeting areas of the brain involved in the control of food intake. Naltrexone is an opioid receptor antagonist that has been associated with reducing the rewarding effects of food and helping to control cravings (monotherapy used to treat alcohol use disorder and opioid use disorder) [14], but its efficacy has not been established for weight management on its own [15,16]. Bupropion is a norepinephrine-dopamine reuptake inhibitor that is associated with stimulating the activity of pro-opiomelanocortin (POMC) cells, which can suppress appetite through central mechanisms [13,17]. The effectiveness of bupropion alone for weight reduction is minimal; however, bupropion and naltrexone together may act to prevent the endogenous negative feedback of POMC cells, allowing for a sustained effect in decreasing appetite [13,16,17].

Expanding awareness of obesity as a chronic disease has led to an increase in the number of obesity treatments [18]. Primary care professionals should understand the available obesity treatments and evidence supporting their use in appropriate patient populations. The goal of this review is to describe the optimal and appropriate use of NB-ER in the treatment of obesity.

## 2 Pharmacokinetics and pharmacodynamics

The extended-release (ER) formulation of NB-ER is designed to minimize peak plasma concentrations, thus reducing the likelihood of adverse effects [13,19]. Naltrexone and bupropion cross the blood-brain barrier to induce their effects on the central nervous system (primarily on POMC neurons and dopaminergic systems) [20]. However, effects of NB-ER cannot be extrapolated to the individual components of naltrexone or bupropion because the ER formulation modifies the dose, absorption, distribution, metabolism, and excretion of each drug, rendering those characteristics different from those of the individual drugs taken together or independently as monotherapies [[21], [22], [23], [24]]. The naltrexone and bupropion formulation may enhance weight reduction and suppression of appetite and cravings, effects that are not seen when either drug is used alone [25]. Preclinical studies in mice have shown that the combined effect of naltrexone and bupropion increases POMC cell firing rates to above that of either treatment alone, contributing to the inhibition of food intake with NB-ER [16,26].

### 2.1 Differences in doses and formulations

Even though naltrexone and bupropion are available separately, neither monotherapy is indicated for the treatment of obesity, taken either together or individually [20,21,24]. Furthermore, naltrexone and bupropion monotherapies are not available in equivalent doses to those used in NB-ER. NB-ER is available as a single-pill combination of 8 mg ER naltrexone and 90 mg ER bupropion to be taken twice daily for obesity [22]. Immediate-release naltrexone monotherapy is available as oral tablets but can also be administered as long-acting intramuscular injections. Naltrexone indicated for the treatment of alcohol or opioid dependence is available from 50 mg to 100 mg daily [27,28]. Bupropion indicated for the treatment of depression or for smoking cessation is available as immediate-release, sustained-release, and ER oral tablets at dosages ranging from 75 mg to 450 mg daily [24,29].

There are important distinctions between the doses and formulations available for NB-ER vs for naltrexone and bupropion monotherapies. The ER formulation of NB-ER was designed for a steady release of both active drugs. The total daily dose is 32 mg (lower than 50 mg immediate-release naltrexone) and 360 mg (higher than the 150 mg or 300 mg ER bupropion), respectively, for naltrexone and bupropion [21,23]. Patients receiving NB-ER begin treatment with a titrated dosing schedule (Table 1) [11]. Starting in week 1, patients receive 1 tablet (8 mg ER naltrexone/90 mg ER bupropion) per day to be taken in the morning, escalate to 2 tablets per day (1 in the morning and 1 before dinner) in week 2, 3 tablets per day (2 in the morning and 1 before dinner) in week 3, and the target dose of 4 tablets per day (2 in the morning and 2 before dinner) in week 4 and through the remainder of treatment [11]. This dose titration and formulation help to maximize efficacy while reducing the risk of adverse events (AEs). Additionally, clinical experience suggests the twice-daily formulation may be particularly helpful in controlling hunger and cravings in the evening, a period of time that is often challenging for people with obesity who have lost weight. Conversely, bupropion and naltrexone monotherapies are generally prescribed once daily in the morning.

**Table 1:** NB-ER titrated dosing schedule at initiation.

	Morning dose	Evening dose	Total daily dose^b^
Week 1	1 tablet^a^	None	8 mg ER naltrexone/90 mg ER bupropion
Week 2	1 tablet	1 tablet	16 mg ER naltrexone/180 mg ER bupropion
Week 3	2 tablets	1 tablet	24 mg ER naltrexone/270 mg ER bupropion
Week 4+	2 tablets	2 tablets	32 mg ER naltrexone/360 mg ER bupropion

a Each tablet contains 8 mg ER naltrexone and 90 mg ER bupropion.

b In patients with moderate or severe renal impairment and in those with moderate hepatic impairment, the maximum recommended daily dose for NB-ER is 2 tablets (1 tablet each morning and evening). NB-ER is not recommended for use in patients with end-stage kidney disease or in patients with severe hepatic impairment. ER, extended release; NB-ER, fixed-dose, extended-release combination of naltrexone and bupropion.

Despite the dosages commonly used for either monotherapy, results from the phase 3 COR-I trial found that 32 mg of naltrexone and 360 mg of ER bupropion per day (8 mg ER naltrexone and 90 mg ER bupropion per pill) led to a significantly greater percentage of body weight reduction and improvements in cardiometabolic risk factors, such as cholesterol and hemoglobin A1C, vs placebo [30].

## 3 Efficacy

Results from phase 3 trials helped to further establish the weight-reduction effects of NB-ER. Contrave Obesity Research (COR)-I was a randomized, double-blind, placebo-controlled, phase 3 trial of NB-ER (16 mg and 32 mg of ER naltrexone and 360 mg ER bupropion) vs placebo in patients with uncomplicated obesity (BMI, 30–45 kg/m^2^) and controlled hypertension and/or dyslipidemia [30]. All participants in each study group were instructed to follow a hypocaloric diet (500 kcal/day deficit) and were given advice on lifestyle modification (eg, increase physical activity to 90 min/week) [30]. At 56 weeks, NB-ER 16 mg was associated with 5.0 % weight reduction and NB-ER 32 mg was associated with 6.1 % weight reduction vs 1.3 % for placebo [30]. Additionally, NB-ER was found to potentially improve control of eating and responses to food cravings, important barriers to diet adherence.

The Contrave Obesity Research-Behavior Modification (COR-BMOD) trial aimed to expand these findings by adding an intensive program focusing on a reduced-calorie eating plan, increased physical activity, and behavior modification to treatment with NB-ER or placebo. COR-BMOD was a multicenter, randomized, double-blind, placebo-controlled, phase 3 trial of NB-ER in conjunction with an intensive behavior modification lifestyle plan in patients with obesity or a BMI of 27–45 kg/m^2^ with controlled hypertension and/or dyslipidemia [31]. The behavioral modification lifestyle plan included dietary changes of a caloric-restriction diet based on initial weight, instructions to count calories, record food intake, and consume a balanced diet that provided ≥15 % of energy from protein, ≤30 % energy from fat, with the remainder sourced from carbohydrates. During the first 6 months, patients were encouraged to gradually increase planned physical activity to 180 min/week (eg, brisk walking), engage in strength training, and record daily activity [31]. Participants were further instructed to increase activity to 360 min/week during months 7–12. In addition to intensive calorie reduction and physical activity lifestyle modifications, behavioral therapy to cover skills required for maintaining lost weight (eg, meal planning, stimulus control, slow eating) were delivered in group therapy sessions.

At 56 weeks, NB-ER with behavioral modification was associated with significantly greater weight reduction than the intensive lifestyle plan (placebo) alone (9.3 % vs 5.1 %) in the intention-to-treat (ITT) cohort and completer cohort (11.5 % vs 7.3 %) [31]. Additionally, a greater proportion of patients treated with NB-ER than those treated with placebo had ≥5 % reduction of their body weight in both the ITT (66.4 % vs 42.5 %) and completer (80.4 % vs 60.4 %) populations, and a greater proportion of patients treated with NB-ER vs placebo had 10 % and 15 % reductions in body weight [31].

COR-II was a randomized, parallel-arm, double-blind, placebo-controlled, phase 3 trial in patients with obesity or a BMI of 27–45 kg/m^2^ with controlled hypertension and/or dyslipidemia [32]. Similar to COR-I, study participants in both groups were instructed on lifestyle modifications (ie, calorie restriction [500 kcal per day deficit], increased physical activity) throughout follow-up [32]. At 56 weeks, patients achieved 6.4 % body weight reduction with NB-ER vs 1.2 % with placebo in the ITT cohort and 8.2 % vs 1.4 % body weight reduction, respectively, in the completer cohort [32]. A significantly greater proportion of patients taking NB-ER vs placebo achieved ≥5 %, ≥10 %, and ≥15 % body weight reduction in both the ITT population and in the completer population at weeks 28 and 56 [32]. The observed weight reduction and the proportions of patients achieving ≥5 % weight reduction with NB-ER were further supported by findings from the COR-DM, IGNITE, and LIGHT trials [[33], [34], [35]]. Finally, a pooled analysis of the phase 3 COR trials evaluated early weight reduction of ≥5 % as a predictor of long-term weight reduction with NB-ER [36]. Patients with ≥5 % reduction in body weight at 16 weeks were considered “responders” to the medication, and these patients went on to have a mean body weight reduction of 11.7 % by 56 weeks [36]. Overall, at week 56, 85 % of these early responders had ≥5 % reduction, with 57 % having ≥10 %, and 32 % having ≥15 % reductions in body weight [36].

## 4 Safety

Although the safety of NB-ER has been established in the treatment of patients with obesity, the safety of combined bupropion and naltrexone monotherapy for the purposes of chronic weight management has not. In a clinical trial of patients in the indicated treatment population of naltrexone for alcohol use disorder, the most common (≥2 %) treatment-emergent AEs (TEAEs) with naltrexone monotherapy were sleep disturbances, anxiety, dizziness, fatigue, headache, insomnia, nausea, nervousness, somnolence, and vomiting [21]. Of note, when elevated dosages (300 mg/day) of naltrexone monotherapy were used in patients with obesity, reversible elevations in hepatic transaminase were also observed [21]. Similarly, bupropion monotherapy is approved for depression and smoking cessation, but not for chronic weight management [23,24]. The most common TEAEs (≥5 %) reported with bupropion are anxiety, arthralgia, constipation, dizziness, dry mouth, insomnia, nausea, and nervous disturbances [23]. The unique formulation of NB-ER aims to mitigate the incidence, duration, and severity of AEs. The most common TEAEs (≥5 %) with NB-ER were nausea, constipation, headaches, dizziness, fatigue, vomiting, and dry mouth [26]. Many of these symptoms have been shown to decrease over time as patients develop increased tolerance [26]. A post hoc analysis of 6 NB-ER phase 3 trials (COR-BMOD, COR-DM, COR-I, COR-II, IGNITE, and LIGHT) reported that 16.0 % of patients receiving NB-ER and 17.4 % of patients receiving placebo experienced serious AEs [37]. In the phase 3 trials, NB-ER increased baseline systolic and diastolic blood pressure by 1 mm Hg from week 4 to week 8. However, blood pressure values reverted to baseline by week 12, and there was an average decrease of systolic and diastolic blood pressure of 1 mm Hg between weeks 24–56 compared with baseline. Patients receiving placebo had 2–3 mm Hg decreases in systolic and diastolic blood pressure below baseline throughout the same time points. These changes were correlated with weight reduction [11]. Guidelines recommend regular monitoring of blood pressure and heart rate in patients taking NB-ER, especially among those at risk for cardiovascular disease [11]. There were no significant differences between placebo and NB-ER groups in the proportion of patients meeting the criteria for psychiatric events [31]. A separate pooled analysis found that psychiatric AEs were less common with NB-ER than with placebo [38]. Due to increased risk of seizure with higher doses of bupropion, risk may be minimized by adhering to the recommended dose titration and twice-daily dosing schedule and avoiding high-fat meals (≥50 % fat [39]; low-fat foods have <3 g of fat per 100 calories), and the tablets should not be split, crushed, or chewed [11]. Additionally, NB-ER is contraindicated in patients with uncontrolled high blood pressure, history of seizures or seizure disorders, anorexia nervosa or bulimia, drug or alcohol withdrawal or chronic opioid use, or in patients with known allergy to NB-ER ingredients [11]. NB-ER is also contraindicated for coadministration with other bupropion-containing products or concomitant monoamine oxidase inhibitors [11]. Furthermore, NB-ER is contraindicated during pregnancy and is not recommended for nursing mothers [22]. Treatment with FDA-approved NB-ER should focus on appropriate patients without contraindications and in subpopulations that are the most likely to benefit from treatment.

## 5 Appropriate use in specific subpopulations of patients with obesity

Studies have also supported the use of NB-ER in various subpopulations of patients with obesity. The American Gastroenterological Association suggests the use of NB-ER in weight-loss interventions for patients with obesity who are attempting to quit smoking and in patients with depression [40]. Results from a 24-week, open-label study found that NB-ER treatment may be associated with sustained nicotine abstinence with no significant increase in body weight from baseline in patients with obesity who stopped smoking after starting NB-ER [41]. Among patients attempting to quit smoking with the use of NB-ER and behavior modification, continuous tobacco-use abstinence (biochemically verified) was self-reported in 48 % of patients for weeks 4–12 and 41 % for weeks 4–24 in the ITT cohort; in the completer cohort, self-reported continuous abstinence rates were 52 % for weeks 4–12 and 59 % for weeks 4–24 [41]. Importantly, patients should also be counseled on and monitored for signs of neuropsychiatric AEs, such as changes in mood, anxiety, and suicidal ideation, which have occurred in some patients taking bupropion for smoking cessation [33]. Thus, NB-ER may be associated with an amelioration of nicotine withdrawal as well as the prevention of post–smoking cessation weight gain in patients with obesity who are trying to stop smoking [41].

For patients with obesity and depression, there is considerable evidence supporting the use of NB-ER for weight reduction [38,[42], [43], [44]]. Treatment with NB-ER leads to changes in brain activity by blunting hypothalamic activity and increasing activity in areas involved in self-control and awareness that ultimately reduce reactivity to food cues and emotional eating [10,45]. An open-label, uncontrolled study found that NB-ER may specifically reduce binge-eating in patients with depression [42]. Use of antidepressants with NB-ER is generally well tolerated in patients with overweight or obesity, and NB-ER promotes weight reduction regardless of antidepressant use [44].

Type 2 diabetes presents a significant challenge in achieving weight reduction among individuals with obesity. This clinical observation is attributed to a complex multifactorial etiology consisting of a multitude of disease and patient factors [46]. Results from COR-DM, a 56-week, double-blind, placebo-controlled study that evaluated the effect of NB-ER on body weight in patients with obesity and with type 2 diabetes revealed significant reduction in body weight (≥5 % weight loss) was achieved in 53 % of patients treated with NB-ER versus in 24 % of placebo-treated patients [33]. The overall efficacy observed in COR-DM was lower than that observed in the phase 3 COR-I and COR-BMOD studies where 62 % and 80 % of patients treated with NB-ER achieved ≥5 % weight loss, respectively [30,31]. These observations are also consistent with other treatments for patients with obesity, such as tirzepatide. In the SURMOUNT 2 (patients with obesity and type 2 diabetes) vs SURMOUNT 1 (patients with obesity without diabetes) trials, there was a lower average weight reduction of 12.8 % vs 19.5 % with the 10-mg dose and 14.7 % vs 20.9 % with the 15-mg dose at 72 weeks [47,48]. However, patients with diabetes treated with NB-ER did have significant improvements in reduced fasting insulin (NB-ER, −13.5 % [95 % confidence interval, −19.7 % to −6.8 %] change at week 56 from baseline value of 15.1 ± 1.9 μIU/mL; placebo −10.4 [95 % confidence interval, −18.8 to −1.1] change at week 56 from baseline value of 13.8 ± 1.9 μIU/mL) and HbA_1c_ (NB-ER, −0.6 ± 0.1 % change at week 56 from baseline value of 8.0 ± 0.8 %; placebo −0.1 ± 0.1 % change at week 56 from baseline value of 8.0 ± 0.9 %) levels over time [33].

Consistent with other trials evaluating antiobesity treatments [47,49,50], the COR trials enrolled predominantly female populations (COR-I, 85 %; COR-II, 84.6 %; COR-DM, 58 %; COR-BMOD, 89 %) [[30], [31], [32], [33]]. Therefore, sex-specific difference on response to weight loss will require further evaluation.

Furthermore, patients with obesity present with diverse phenotypes, which may account for their varied responses to obesity interventions, highlighting the need for individualized treatments [51]. Although there are no established predictors for weight reduction, pathophysiologic phenotypes have been identified that can each be targeted differently to enhance the effects of weight-loss interventions [51]. These identified phenotypes may include patients who consume excessive calories because of an inability to determine their fullness; those who eat due to negative mood, cravings, or reward-seeking behaviors; those who eat because they do not feel full for long enough and have excessive gastric emptying; or those who have reduced resting energy expenditure, inadequate physical activity, and lower muscle mass [51,52]. Understanding these different phenotypes can help primary care professionals decide on a course of treatment that is the most likely to be effective in these subpopulations of patients with obesity. Results from a phase 3, pragmatic, real-world trial in 312 patients over 12 months identified potential FDA-approved obesity management medications for each phenotype based on the respective mechanisms of action for each treatment and found phenotype-guided treatment to improve weight-loss outcomes [51]. In this study, phentermine-topiramate ER was used for patients with an abnormal satiation phenotype [51,52], NB-ER for patients with obesity and an emotional eating phenotype [[51], [52], [53]], liraglutide for patients with abnormal postprandial satiety [51,52], and low-dose phentermine for patients with abnormal resting energy expenditure [51,52].

Socioeconomic factors and long-term costs associated with obesity treatments, in addition to the identified phenotypes, also contribute to the importance of appropriately using NB-ER and other treatment options for patients with obesity [54]. The current costs of medications such as tirzepatide ($550–$1118/month [55,56]), semaglutide ($717–$1225/month), and liraglutide ($761–$1281/month) can impose a financial burden on patients. Furthermore, patients will not respond uniformly to these treatments, with subpopulations being potentially better suited to one option than to another. Health care professionals may prefer to prescribe generics to help alleviate cost, but the difference in cost between NB-ER and generic naltrexone and bupropion is minimal [22,54]. The cost of NB-ER (Contrave) can vary, but for patients without insurance, the retail price for a 30-day supply of Contrave is approximately $350 to $400 [22,54]. However, many patients can significantly reduce this cost to $99 per month through currently available savings programs and discounts, more in line with the generic components (bupropion, $15–$20 average monthly out-of-pocket cost; naltrexone monthly cost, $27–$60); though as noted, the efficacy and safety of these monotherapies for the treatment of patients with obesity has not been established and would therefore be considered off-label for use in the treatment of obesity [22].

When considering NB-ER for obesity treatment, it is important to note how this therapy differs from available obesity treatments such as glucagon-like peptide 1 (GLP-1) receptor agonists [54]. NB-ER is an oral medication, whereas GLP-1 receptor agonists are often administered via injection [54,57]. GLP-1 receptor agonists may lead to injection-related reactions, whereas NB-ER will not [11,57]. Conversely, the oral administration of NB-ER contraindicates its use with high-fat meals [58]. The mechanisms of these different treatments also vary, with NB-ER reducing appetite and cravings, while GLP-1 receptor agonists increase insulin secretion to promote satiety and reduce food intake [54]. GLP-1 receptor agonists also require refrigerated shipping and storage (2 °C–8 °C), which may pose a challenge for both pharmacies and patients [15,29]. Among patients with obesity, GLP-1 receptor agonists tend to result in greater weight reduction compared with NB-ER [59]. In the SCALE Obesity and Prediabetes trial, liraglutide was associated with a mean 8 % weight reduction at 52 weeks from baseline [60]. In the STEP 1 trial, semaglutide was associated with a mean 14.9 % weight reduction at 68 weeks from baseline [49]. In the SURMOUNT-1 trial, the GLP-1 and glucose-dependent insulinotropic polypeptide agonist tirzepatide (15 mg) was associated with a mean 20.9 % weight reduction at 72 weeks from baseline [47]. Treatment options for patients with obesity are associated with gastrointestinal side effects, though GLP-1 receptor agonists can also cause pancreatitis, whereas NB-ER can lead to seizures [11,54,57]. Therefore, the best treatment option will depend on the history, characteristics, and treatment goals of the patient and should be carefully discussed by the patient and their health care team. Once an effective treatment course is established, treatment should be continued long-term, consistent with the management of other chronic diseases, to promote long-term weight maintenance [12].

## 6 Conclusions

Obesity is a chronic disease that is most effectively addressed through individualized treatment. Unlike the monotherapy versions of its component drugs, NB-ER is optimized for the treatment of patients with obesity, with efficacy and safety established through a series of clinical trials. Off-label use of the individual components, alone or together, is not supported by clinical trial evidence or real-world studies. Among patients with obesity, there are specific subpopulations that may be most likely to benefit from treatment with NB-ER. Decisions to use NB-ER in patients with obesity should take into consideration adiposity-related complications and patient-specific characteristics for optimal and sustained weight reduction.

Three bulleted key takeaway clinical messages:

1. Obesity treatment should be personalized to a patient's individual characteristics and adiposity-related complications.
2. NB-ER is a unique fixed-dose, extended-release formulation of naltrexone and bupropion that targets both homeostatic and hedonic brain pathways that may work together to reduce weight compared with either monotherapy alone or taken in combination.
3. As an adjunct to behavior modification (eg, reduced caloric intake and increased physical activity), the unique formulation of NB-ER is optimized for the safe and effective treatment of patients with obesity.

## Informed consent statement

Not applicable.

## Author contribution

**Ethan Lazarus:** Conceptualization, methodology, writing—original draft, writing—review and editing.

## Institutional review board statement

Not applicable.

## Data availability statement

Not applicable.

## Ethical adherence and ethical review

Responsibility for editorial decisions and peer review for this article was delegated to non-author editors or associate editors.

## Declaration of artificial intelligence (AI) and AI-assisted technologies

The author did not use AI during the preparation of this manuscript.

## Funding

This work was sponsored by Currax Pharmaceuticals, LLC.

## Declaration of competing interest

The authors declare the following financial interests/personal relationships which may be considered as potential competing interests: Ethan Lazarus reports financial support was provided by Currax Pharmaceuticals LLC. Ethan Lazarus reports a relationship with Currax Pharmaceuticals LLC that includes: speaking and lecture fees. Ethan Lazarus reports a relationship with Lilly that includes: consulting or advisory and speaking and lecture fees. Ethan Lazarus reports a relationship with Novo Nordisk Inc that includes: speaking and lecture fees. Ethan Lazarus reports a relationship with Boehringer Ingelheim that includes: consulting or advisory. Ethan Lazarus reports a relationship with Obesity Medicine Association that includes: board membership. If there are other authors, they declare that they have no known competing financial interests or personal relationships that could have appeared to influence the work reported in this paper.
